# Determinants of Agricultural Pesticide Concentrations in Carpet Dust

**DOI:** 10.1289/ehp.1002532

**Published:** 2011-02-17

**Authors:** Robert B. Gunier, Mary H. Ward, Matthew Airola, Erin M. Bell, Joanne Colt, Marcia Nishioka, Patricia A. Buffler, Peggy Reynolds, Rudolph P. Rull, Andrew Hertz, Catherine Metayer, John R. Nuckols

**Affiliations:** 1 Cancer Prevention Institute of California, Berkeley, California, USA; 2 Division of Cancer Epidemiology and Genetics, National Cancer Institute, National Institutes of Health, Department of Health and Human Services, Bethesda, Maryland, USA; 3 Westat, Inc., Rockville, Maryland, USA; 4 University at Albany, State University of New York, Albany, New York, USA; 5 Battelle Memorial Institute, Columbus, Ohio, USA; 6 School of Public Health, University of California–Berkeley, Berkeley, California, USA; 7 Department of Environmental and Radiological Health Sciences, Colorado State University, Fort Collins, Colorado, USA

**Keywords:** agriculture, dust, exposure, GIS, pesticides

## Abstract

Background: Residential proximity to agricultural pesticide applications has been used as a surrogate for exposure in epidemiologic studies, although little is known about the relationship with levels of pesticides in homes.

Objective: We identified determinants of concentrations of agricultural pesticides in dust.

Methods: We collected samples of carpet dust and mapped crops within 1,250 m of 89 residences in California. We measured concentrations of seven pesticides used extensively in agriculture (carbaryl, chlorpyrifos, chlorthal-dimethyl, diazinon, iprodione, phosmet, and simazine). We estimated use of agricultural pesticides near residences from a statewide database alone and by linking the database with crop maps. We calculated the density of pesticide use within 500 and 1,250 m of residences for 180, 365, and 730 days before collection of dust and evaluated relationships between agricultural pesticide use estimates and pesticide concentrations in carpet dust.

Results: For five of the seven pesticides evaluated, residences with use of agricultural pesticides within 1,250 m during the previous 365 days had significantly higher concentrations of pesticides than did residences with no nearby use. The highest correlation with concentrations of pesticides was generally for use reported within 1,250 m of the residence and 730 days before sample collection. Regression models that also accounted for occupational and home use of pesticides explained only a modest amount of the variability in pesticide concentrations (4–28%).

Conclusions: Agricultural pesticide use near residences was a significant determinant of concentrations of pesticides in carpet dust for five of seven pesticides evaluated.

Residential proximity to reported use of agricultural pesticides has been used to estimate exposure in health studies. Epidemiologic studies have observed an association between residential proximity to use of agricultural pesticides and fetal death (Bell et al. 2001), neural tube defects (Rull et al. 2006), autism (Roberts et al. 2007), Parkinson’s disease (Costello et al. 2009), and childhood cancer (Carozza et al. 2008). However, other epidemiologic studies have not observed an association between proximity to reported use of agricultural pesticides and breast cancer (Reynolds 2005a) and childhood cancer (Reynolds 2005b) or observed an association between proximity to moderate but not high reported use and childhood acute lymphoblastic leukemia (Rull 2009). Uncertainty of exposure is a limitation of previous health studies, and more work is needed to better understand the relationship between residential proximity to reported use of agricultural pesticides and individual exposures.

California is the largest agricultural state in the United States and applies > 86 million kilograms of pesticides per year [California Department of Pesticide Regulation (CDPR) 2008]. California has a mandatory agricultural pesticide use reporting program [California Pesticide Use Reporting (CPUR) database] for all commercial pesticide applications, including the date, location, active ingredients, amount applied, and crop treated. In previous work, we used a geographic information system (GIS) to link CPUR and land use data to estimate pesticide use near residences. We found that the addition of crop maps resulted in substantial differences in estimated density of pesticide use within 500 m of residences, indicating a need to evaluate whether this difference could affect potential exposure (Nuckols et al. 2007).

In the present study, we collected and analyzed carpet dust samples from homes in agricultural areas of California to evaluate the relationship with the use of agricultural pesticides ascertained from the CPUR database. Carpet dust is a good environmental medium for assessing long-term exposure in the home because pesticides and other chemicals persist indoors, where they are protected from degradation by sunlight, moisture, and microorganisms (Lioy et al. 2002; Roberts et al. 2009). Concentrations of metals, pesticides, and persistent organic pollutants in dust have been used as indicators of exposure in previous studies (Colt et al. 2005; Rudel et al. 2001; Ward et al. 2009; Whitehead et al. 2009). Previous studies have reported that concentrations of agricultural pesticides in carpet dust are higher in residences closer to treated fields and in farm homes (Curwin et al. 2005; Fenske et al. 2002; Harnly et al. 2009; Lu et al. 2000; Obendorf et al. 2006; Simcox et al. 1995; Ward et al. 2006). We build on our previous work by comparing estimates of residential proximity and reported use of agricultural pesticides from CPUR data alone versus CPUR data integrated with crop maps and concentrations of pesticides in carpet dust samples from homes in agricultural areas of California. The purpose of our study was to identify determinants of concentrations of agricultural pesticides in carpet dust.

## Materials and Methods

*Study population and sample collection.* We included 89 residences from two studies conducted in northern and central California in our analyses that met the criterion of having a minimum of 25% of the land area within 500 m of the residence in agricultural fields as determined by existing land use maps. We conducted a land use survey to identify agricultural crops within a radius of 1,250 m around each residence within an average of 14 days after the collection of the dust sample [interquartile range (IQR), 8–22 days]. We collected latitude and longitude coordinates at the front door of each home using a global positioning system device to locate the residences for GIS analysis. All homes had a carpet or area rug that was at least 1 m^2^ in area, the minimum area needed for carpet dust sampling. Participants completed an interview at the time of dust collection about home and garden pesticide use and occupations held by people living in the residence during the previous 12 months. Our analysis included 68 residences from the Northern California Childhood Leukemia Study, a population-based case–control study in 17 counties in the San Francisco Bay area and 18 counties in the Central Valley (Chang et al. 2006; Ward et al. 2009). An additional 21 residences were included from the Agricultural Pesticide Study in Fresno, California, designed to evaluate determinants of agricultural pesticides in homes.

A dust sample was collected from each residence between December 2001 and March 2006 as described previously (Colt et al. 2008). Samples were collected using a high-volume surface sampler, a specially designed vacuum cleaner that collects particles > 5 μm in diameter (HVS3; Cascade Sampling Systems, Bend, OR). For the leukemia study participants, the dust sample was taken in a room other than his or her bedroom, and where the child had spent the most time if there was an eligible carpet or rug. Otherwise, another room where the child spent time with an eligible carpet was selected. Most samples were taken in the living room or family room. For the Fresno study participants, the sample room was selected from the rooms located on the side of the home facing crops. If the first room did not contain an eligible carpet or area rug, another room was selected. Interview staff collected dust until the collection bottle contained mass of dust roughly equivalent to 20 mL volume. The median area sampled was 2.4 m^2^, and the IQR was 2.2–3.1 m^2^. The median mass of dust collected was 3.5 g (IQR, 1.8–7.4 g).

*Pesticide selection and laboratory analysis.* Among the 34 pesticides measured in the dust samples, we selected seven for this analysis that were *a*) frequently detected in carpet dust samples from this study population (> 33% of residences), *b*) used extensively (> 100,000 kg/year) in California between 2000 and 2006, and *c*) used primarily in agriculture (> 50% of sales in California reported in the CPUR database). Seven pesticides met all three criteria: the insecticides carbaryl, chlorpyrifos, diazinon, and phosmet; the herbicides chlorthal-dimethyl (also called dacthal or DCPA) and simazine; and the fungicide iprodione (Table 1).

**Table 1:** Quantity sold, reported agricultural use in California, and detections and concentrations of selected pesticides in carpet dust samples from 89 homes.

Table 1. Quantity sold, reported agricultural use in California, and detections and concentrations of selected pesticides in carpet dust samples from 89 homes.												
Pesticide (type)		Sales*a *(kg)		Agricultural use*b *(%)		DL (ng/g)		Detects (%)		GM (GSD) (ng/g)		Median (IQR)*c *(ng/g)
Carbaryl (I)		190,938		55		2		84		24 (9)		26 (10–113)
Chlorpyrifos (I)		986,181		82		5		96		48 (4)		46 (25–116)
Chlorthal-dimethyl (H)		136,225		76		1		44		1 (4)		3 (2–10)
Diazinon (I)		402,701		73		2		93		30 (7)		23 (9–86)
Iprodione (F)		156,698		82		20		34		19 (3)		58 (45–100)
Phosmet (I)		312,085		80		25		37		27 (4)		102 (33–441)
Simazine (H)		498,876		61		2		92		25 (5)		21 (11–58)
Abbreviations: DL, detection limit; F, fungicide; GM, geometric mean; GSD, geometric standard deviation; H, herbicide; I, insecticide. **a**Average annual statewide sales in California from 2000 through 2006 (CDPR 2009). **b**Percentage of statewide sales reported as agricultural in CPUR from 2000 through 2006 (CDPR 2008). **c**Distribution among residences with pesticide concentrations above the detection limit.												

Detailed laboratory methods have been published elsewhere (Colt et al. 2008). Briefly, for the seven pesticides included in these analyses, we sieved carpet dust samples (< 150 μm) and extracted approximately 0.5 g of dust in 12 mL hexane:acetone solvent, which was then centrifuged and concentrated to 1 mL. We quantified concentrations of pesticides using gas chromatography/mass spectrometry in the multiple ion detection mode. Quality control samples included duplicates, the same duplicates spiked with 250 ng of each analyte, and a solvent method blank. We spiked ^13^C-labeled surrogate recovery standards (SRSs) into all samples before extraction to aid in identification and as a check on method performance. We analyzed an 8-point calibration curve, spanning the range of 2–750 ng/mL for analytes and 10–300 ng/mL for SRSs, plus an instrument blank, concurrently with each sample set. Detection limits for the seven pesticides ranged from 1 to 25 ng/g of dust (Table 1). Duplicate samples had average relative percent differences of 10–30%. Mean sample recoveries for spiked samples ranged from 85% to 118%, and SRS recoveries averaged between 82% and 111% in quality control samples. Results were similar using SRS-corrected and uncorrected concentrations (Pearson correlation coefficients > 0.95); therefore, we report uncorrected concentrations. For one carpet dust sample, the concentration of iprodione could not be quantified because of interfering substances.

*Geographic-based estimates of agricultural pesticide use.* We used the following CPUR data from 2000 through 2006 to estimate the use of agricultural pesticides around the home: pounds of active ingredient applied, crop treated, acres treated, and application date and location. The location of pesticide application is reported in the CPUR database for each section (~ 1.6 × 1.6 km) defined by the Public Land Survey System (Cal-Atlas 2011). We used a GIS (ArcGIS; ESRI, Redlands, CA)to calculate the density of pesticide use in kilograms per square kilometer using the CPUR data alone (“CPUR method”) and the CPUR data combined with agricultural land use maps (“CROP method”) for six combinations of time (during the 180, 365, and 730 days before carpet dust sample collection) and distance from the latitude/longitude coordinates of the residence (500- and 1,250-m radius). We selected these distances because 500 m has been used in most previous epidemiologic studies using CPUR data (Costello et al. 2009; Ritz et al. 2009; Roberts et al. 2007; Rull et al. 2006), and 1,250 m represents the area for which we mapped crops around the residences, as well as an upper range of the distance associated with pesticide drift (Teske et al. 2002; Woods et al. 2001). We selected the time periods to coincide with the self-reported interview data on pesticide use (1 year), the upper limit that we judged the crop maps to be representative of actual cropping patterns (2 years), and the time period most associated with concentrations of pesticides in house dust (180 days) in the previous study that evaluated the relationship with CPUR data (Harnly et al. 2009).

**Figure 1:**
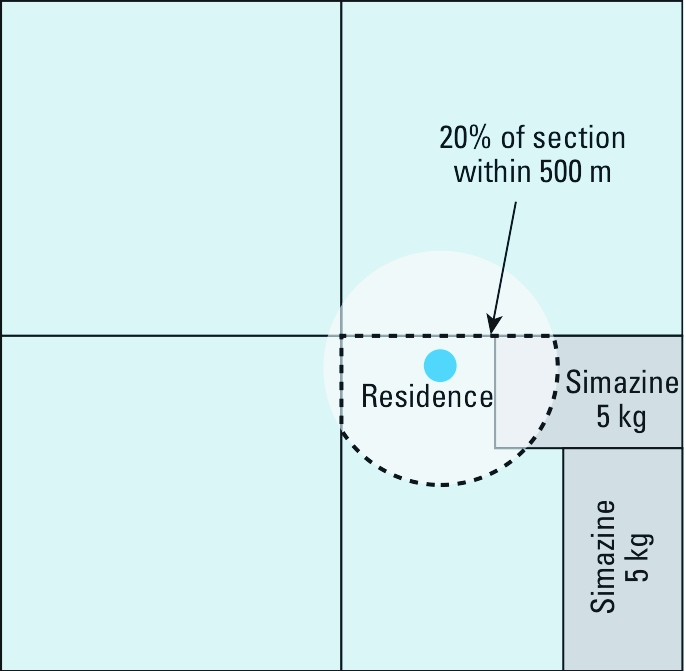
Metric used to estimate density of pesticide use (kg/km^2^) within a circular buffer around a home using CPUR data only, where 10 kg (5 kg + 5 kg) simazine was applied to the section in the lower right quadrant with 20% of its land area within 500 m of the residence. The resulting simazine use density was (10 kg × 0.2)/0.785 km^2^ = 2.5 kg/km^2^ by the CPUR method.

*CPUR method.* A detailed description of the equations used to calculate density of pesticide use for both the CPUR and CROP methods has been published previously (Nuckols et al. 2007). Briefly, for each pesticide we calculated the density of pesticide use by estimating the proportion of land area for each section within a given distance of a residence (GIS buffer), multiplying the amount (kilograms) of pesticide active ingredient applied to each section during the time period by the proportion of area within the buffer, summing the area-weighted kilograms for all sections intersected by the buffer, and dividing by the area of the buffer (0.785 km^2^ for a 500-m buffer and 4.91 km^2^ for a 1,250-m buffer). Figure 1 illustrates a sample CPUR metric calculation.

**Figure 2:**
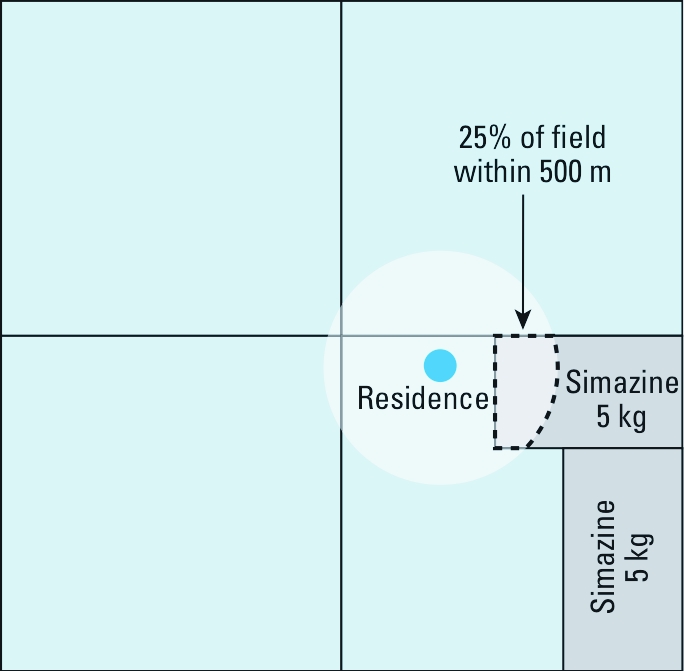
Metric used to estimate density of pesticide use (kg/km^2^) within a circular buffer around a home using CPUR data linked to crop maps (CROP), where 5 kg simazine was applied to a field in the section in the lower right quadrant with 25% of its area within 500 m and 5 kg simazine was applied to another field with 0% of its area within 500 m. The resulting simazine use density was (5 kg × 0.25 + 5 kg × 0)/0.785 km^2^ = 1.6 kg/km^2^ by the CROP method.

The CROP method uses both CPUR data and land use maps created for each residence. The primary difference between the two exposure metrics is that the CROP metric is a function of the area of crops on which the pesticide is used within the buffer, whereas the CPUR metric is a function of the area of the sections within the same buffer without regard to where the treated crops are located within the section. For each crop field from our land use survey that was within the residence buffer, we estimated a crop-specific pesticide application rate for each pesticide used in each section, using data from the CPUR on crop and acres treated. For each section that intersected the residence buffer, we divided the amount of the pesticide applied to each crop during the time period of interest by the total area of the crop treated with the pesticide in the section. We then multiplied this crop-specific section application rate by the section’s crop area within the buffer based on our crop maps to obtain the CROP-weighted kilograms for that section. If only a portion of the total crop area was treated and the CROP weighted application amount exceeded the total kilograms applied in the section, we used the total kilograms applied in the section instead. We then summed the CROP weighted kilograms for all sections within the buffer and divided by the area of the buffer to compute the CROP metric. Figure 2 illustrates a sample CROP metric calculation.

*Interview data.* At the time of dust collection, we asked participants about treatments for pests in and around the home and garden during the previous 12 months. Specifically, we asked about pesticide treatments for ants and cockroaches, bees and wasps, fleas and ticks, flies and mosquitoes, plant diseases, and lawn and garden weeds. We also asked about treatment for any other indoor pests and about professional pesticide treatments inside and outside the home, including the lawn and garden. We assessed potential occupational take-home exposures by asking participants whether anyone in the household worked during the previous 12 months in the following occupations: farmworker or ranch worker; gardener, landscaper, nursery worker, or groundskeeper; agricultural packer; or worker who handles, formulates, or mixes pesticides. We used interview data on use of pesticides at home and work in regression models to adjust for sources other than nearby agricultural use.

*Statistical analyses.* Because the pesticide concentrations were not normally distributed, we used nonparametric methods for initial comparisons. We used the Wilcoxon signed rank test to compare the distributions of paired densities of pesticide use (kilograms per square kilometer) using the CPUR and CROP metrics and Spearman rank correlation coefficients (ρ_s_) to assess the association between the two metrics and concentrations of pesticides (nanograms per gram) in dust. We used the Wilcoxon rank sum test to compare the distribution of concentrations of pesticides in carpet dust for residences near use of agricultural pesticides and those with no use of agricultural pesticides nearby using both exposure metrics, and also to compare residences with self-reported home or occupational pesticide use and those without self-reported use.

We used both linear and Tobit regression to model the log-transformed concentrations of pesticides. For the linear regression models, we assigned samples with concentrations below the limit of detection one-half the detection limit. Tobit regression offers an unbiased approach for analyzing measurement data with detection limits which is important when a large proportion of samples are below the limit of detection (Lubin et al. 2004). We created separate models for each pesticide and metric of agricultural pesticide use for a distance of 1,250 m and 365 days before dust collection to coincide with the time period of the interview data. Independent variables in the models included the CPUR or CROP metrics, self-reported home and garden use of pesticides by pest treated (use in the preceding 12 months, yes or no), potential occupational pesticide exposure (anyone in the household employed during the preceding 12 months for each occupation, yes or no) and the year (continuous variable from 2001 to 2006, centered by subtracting 2000) and season of dust collection (winter = 1, spring = 2, summer = 3, and fall = 4). We used backward elimination to select variables with *p* ≤ 0.1 to remain in the final models.

This study protocol was approved by the institutional review boards of all participating institutions. All participants gave written informed consent.

## Results

Table 1 presents detection limits, percentage of carpet dust samples with detectable levels, and distribution of concentrations for the seven pesticides. Geometric mean pesticide concentrations ranged from 1 ng/g (the limit of detection for chlorthal-dimethyl) to 48 ng/g (chlorpyrifos). The median density of agricultural pesticide use during the 365 days before collection of dust ranged from 1 kg/km^2^ (carbaryl) to 33 kg/km^2^ (chlorthal-dimethyl) among residences classified as being within 1,250 m of agricultural use according to CPUR or CROP metrics (Table 2). Wilcoxon signed rank tests indicate that the estimated density of pesticide use differed (*p* < 0.01) between CPUR and CROP metrics for chlorpyrifos, diazinon, iprodione, phosmet, and simazine, but not for carbaryl or chlorthal-dimethyl.

**Table 2:** Estimated density of agricultural pesticide use within 1,250 m of residences (*n* = 89) during the 365 days before collection of dust samples.

Table 2. Estimated density of agricultural pesticide use within 1,250 m of residences (*n* = 89) during the 365 days before collection of dust samples.								
Pesticide		Metric		Exposed residences [*n* (%)]		Median (IQR) density of use*a *(kg/km^2^)		Median (IQR) applications near residence*a*
Carbaryl		CPUR		37 (42)		1 (0.2–5)		3 (1–7)
		CROP		24 (27)		1 (0.4–9)		2 (1–5)
Chlorpyrifos*		CPUR		77 (87)		10 (4–21)		13 (4–39)
		CROP		70 (79)		17 (7–45)		9 (4–19)
Chlorthal-dimethyl		CPUR		8 (9)		9 (3–35)		66 (13–92)
		CROP		6 (7)		33 (8–76)		20 (3–36)
Diazinon*		CPUR		42 (47)		3 (0.6–5)		7 (4–23)
		CROP		31 (35)		7 (4–29)		5 (1–10)
Iprodione*		CPUR		58 (66)		2 (0.6–5)		12 (3–19)
		CROP		47 (53)		7 (3–13)		7 (3–18)
Phosmet*		CPUR		46 (52)		7 (1–28)		7 (2–19)
		CROP		36 (40)		13 (3–50)		5 (2–18)
Simazine*		CPUR		56 (63)		5 (2–11)		11 (4–26)
		CROP		48 (54)		13 (5–27)		8 (4–19)
**a**Distribution among residences classified as exposed to agricultural pesticide use within 1,250 m and 365 days based on CPUR or CROP metric. *Significant (*p* < 0.01) difference between rank distributions of CPUR and CROP.								

Table 3 provides Spearman rank correlations (ρ_s_) between estimated density of agricultural pesticide use (kilograms per square kilometer) and pesticide concentrations in carpet dust (nanograms per gram) for the seven pesticides according to distance from the residence (500 or 1,250 m) and time period (180, 365, or 730 days). Carpet dust concentrations and density of use estimates (based on CPUR or CROP) were moderately correlated (ρ_s_ = 0.31–0.51) in a least one distance–time category for all pesticides except carbaryl and diazinon (ρ_s_ = 0.17–0.23). Correlations were stronger with density of use estimated within 1,250 m than within 500 m for all pesticides except simazine, and for density of use within 730 days than for shorter time periods for all pesticides except diazinon, iprodione, and phosmet.

**Table 3:** Spearman rank correlation coefficients (ρ_s_) between density of pesticide use (kg/km^2^) and concentrations of pesticides in carpet dust (ng/g) for 89 residences.

Table 3. Spearman rank correlation coefficients (ρ_s_) between density of pesticide use (kg/km^2^) and concentrations of pesticides in carpet dust (ng/g) for 89 residences.														
				Distance from residence and days before dust collection										
				500 m						1,250 m				
Pesticide		Metric		180 days		365 days		730 days		180 days		365 days		730 days
Carbaryl		CPUR		0.16		0.08		0.14		0.12		0.11		0.19
		CROP		0.10		0.10		0.22*		0.09		0.12		0.23*
Chlorpyrifos		CPUR		0.03		0.18		0.25*		0.11		0.27*		0.31**
		CROP		0.25*		0.23*		0.28**		0.27**		0.28**		0.33**
Chlorthal-dimethyl		CPUR		0.35**		0.35**		0.35**		0.28**		0.31**		0.37**
		CROP		0.30**		0.27**		0.30**		0.36**		0.33**		0.36**
Diazinon		CPUR		0.12		0.13		0.12		0.16		0.10		0.13
		CROP		0.07		0.05		0.07		0.17		0.09		0.14
Iprodione		CPUR		0.21*		0.33**		0.31**		0.28**		0.36**		0.34**
		CROP		0.30**		0.30**		0.34**		0.34**		0.33**		0.34**
Phosmet		CPUR		0.38**		0.34**		0.33**		0.38**		0.37**		0.32**
		CROP		0.31**		0.28**		0.29**		0.40**		0.38**		0.34**
Simazine		CPUR		0.46**		0.43**		0.51**		0.46**		0.46**		0.50**
		CROP		0.50**		0.41**		0.48**		0.49**		0.45**		0.48**
*p < 0.05, **p < 0.01.														

Based on univariate analyses using the Wilcoxon rank sum test, the distributions of concentrations of pesticides were higher in residences where we estimated agricultural pesticide use within 1,250 m and during the previous 365 days compared with those without agricultural use for all pesticides except diazinon, and the difference was significant (*p* < 0.05) for all pesticides except carbaryl and diazinon (Table 4). Median carpet dust concentrations of pesticides in homes classified as being exposed to agricultural use were higher for carbaryl, chlorpyrifos, chlorthal-dimethyl, and simazine when we classified exposure based on the CROP metric compared with the CPUR metric. Chlorpyrifos concentrations were higher in carpet dust samples from the homes of individuals who reported using pesticides for fleas or ticks during the previous year compared with other homes (median, 69 vs. 31 ng/g for 32 and 57 homes, respectively) and in dust samples from households with a person employed as a farmworker or ranch worker during the previous year compared with households without a farmworker (median, 100 vs. 37 ng/g for 12 and 77 homes, respectively). We found no substantial differences (*p* < 0.1) in concentrations of other pesticides among households that reported home and garden pesticide use or households with a member working in a pesticide exposed occupation.

**Table 4:** Univariate analyses of concentrations of pesticides in carpet dust for residences with agricultural, home, or occupational pesticide use compared with those with no use: previous 365 days.

Table 4. Univariate analyses of concentrations of pesticides in carpet dust for residences with agricultural, home, or occupational pesticide use compared with those with no use: previous 365 days.								
Pesticide, explanatory variables*a*		Exposed residences [*n* (%)]		Exposed median (ng/g)		Unexposed median (ng/g)		*p*-Value*b*
Carbaryl								
CPUR								
500 m		19 (21)		44		15		0.4
1,250 m		37 (42)		26		15		0.4
CROP								
500 m		11 (12)		47		17		0.4
1,250 m		24 (27)		30		16		0.4
Chlorpyrifos								
CPUR								
500 m		68 (73)		47		28		0.18
1,250 m		77 (87)		47		19		0.03
CROP								
500 m		48 (54)		58		34		0.03
1,250 m		70 (79)		51		25		0.07
Flea or tick		32 (36)		69		31		0.05
Farmworker or ranch worker		12 (13)		100		37		0.02
Chlorthal-dimethyl								
CPUR								
500 m		4 (5)		26		0.5		0.001
1,250 m		8 (9)		9		0.5		0.005
CROP								
500 m		2 (2)		46		0.5		0.01
1,250 m		6 (7)		15		0.5		0.002
Diazinon								
CPUR								
500 m		29 (33)		18		19		0.1
1,250 m		42 (47)		20		18		0.3
CROP								
500 m		18 (20)		15		23		0.7
1,250 m		31 (35)		18		21		0.4
Iprodione								
CPUR								
500 m		42 (48)		15		10		0.001
1,250 m		58 (66)		10		10		0.008
CROP								
500 m		26 (30)		15		10		0.007
1,250 m		47 (53)		10		10		0.003
Phosmet								
CPUR								
500 m		31 (35)		16		13		0.003
1,250 m		46 (52)		13		13		0.004
CROP								
500 m		18 (20)		14		13		0.03
1,250 m		36 (40)		13		13		0.008
Exterior professional		23 (26)		22		13		0.06
Simazine								
CPUR								
500 m		43 (48)		41		14		0.0001
1,250 m		56 (63)		34		14		0.0006
CROP								
500 m		38 (43)		43		14		0.0002
1,250 m		48 (54)		38		14		0.0001
Pesticide mixer		1 (1)		2,146		21		0.09
**a**Results provided for agricultural pesticide use metrics and for other explanatory variables if *p* ≤ 0.1. **b**Wilcoxon rank sum test compares distribution of those classified as exposed and those classified as unexposed.								

Table 5 provides linear and Tobit regression model estimates of the proportional increase in concentrations of pesticides in carpet dust associated with different sources of exposure. Both the CPUR and CROP metrics were significant determinants (*p* < 0.05) of concentrations of chlorpyrifos, chlorthal-dimethyl, iprodione, phosmet, and simazine. A 2.7-fold increase in agricultural pesticide use (a one-unit change on the natural logarithm scale) resulted in 1.2–3.4 times higher concentrations of these pesticides in carpet dust. The same predictor variables were significant in both linear and Tobit regression models. For pesticides with more than 50% of measurements below the limit of detection (chlorthal-dimethyl, iprodione, and phosmet), associations between dust concentrations and agricultural use were substantially greater based on estimates from Tobit model compared with linear regression models. CPUR and CROP metrics were not significant predictors of diazinon or carbaryl concentrations in carpet dust, but diazinon concentrations were inversely association with year of dust sample collection (*p* < 0.001), and carbaryl concentrations were higher in samples collected during the fall than the summer, the summer than the spring, and the spring than the winter. Having a person in the home that was a farmworker or ranch worker during the previous year was associated (*p* < 0.05) with higher concentrations of chlorpyrifos in carpet dust. Only one person reported handling or mixing pesticides during the previous year, but this was significantly associated (*p* < 0.01) with simazine concentrations in carpet dust. Regression models including CPUR- or CROP-based estimates of agricultural pesticide use and data on self-reported home use of pesticides and potential occupational exposure during the previous year explained a modest proportion of the variability in concentrations of pesticides in dust (coefficient of determination *R*^2^ = 0.04–0.24).

**Table 5:** Proportional increase in concentrations of pesticides in carpet dust from multivariate regression models: previous 365 days, within 1,250 m.

Table 5. Proportional increase in concentrations of pesticides in carpet dust from multivariate regression models: previous 365 days, within 1,250 m.										
		Linear regression results						Tobit regression results		
Pesticide, explanatory variables*a*		Exp(β)		95% CI		*R*^2^		Exp(β)		95% CI
Carbaryl										
Season dust collected		1.51		0.95–2.38		0.04		1.69*		0.99–2.89
Chlorpyrifos										
Ln(CPUR)		1.22*		1.00–1.48		0.19		1.23*		1.01–1.50
Flea or tick		2.01*		1.15–3.53				2.07**		1.18–3.67
Farmworker or ranch worker		2.21*		1.03–4.71				2.25*		1.04–4.90
Ln(CROP)		1.22**		1.05–1.42		0.22		1.23**		1.06–1.44
Flea or tick		1.98**		1.15–3.42				2.05**		1.18–3.56
Farmworker or ranch worker		2.30*		1.09–9.81				2.35*		1.10–5.00
Chlorthal-dimethyl										
Ln(CPUR)		2.04^#^		1.55–2.69		0.24		2.49^#^		1.60–3.94
Year dust collected		1.18		0.96–1.47				1.46		0.99–2.16
Ln(CROP)		1.99^#^		1.54–2.57		0.26		2.36^#^		1.57–3.56
Year dust collected		1.19		0.96–1.46				1.45		0.98–2.14
Diazinon										
Year dust collected		0.56^#^		0.39–0.79		0.14		0.55**		0.38–0.80
Exterior professional		2.56*		1.00–6.61				2.80*		1.04–7.54
Iprodione										
Ln(CPUR)		2.50^#^		1.47–4.26		0.12		9.21**		2.10–40.9
Ln(CROP)		2.05^#^		1.39–3.03		0.14		5.47**		1.85–16.1
Phosmet										
Ln(CPUR)		2.66^#^		1.73–4.10		0.25		7.77^#^		2.57–23.5
Season dust collected		1.43**		1.09–1.86				2.48*		1.14–5.37
Ln(CROP)		2.51^#^		1.74–3.60		0.28		6.36^#^		2.55–16.4
Season dust collected		1.34*		1.03–1.75				2.18*		1.02–4.66
Simazine										
Ln(CPUR)		3.42^#^		1.92–6.05		0.25		3.56^#^		2.14–7.46
Pesticide mixer		36.2**		2.14–608				36.6*		27.8–45.4
Ln(CROP)		2.61^#^		1.63–4.18		0.24		2.71^#^		1.75–4.90
Pesticide mixer		45.2**		2.66–765				46.2**		37.3–55.1
Abbreviations: CI, confidence interval; Exp(β), exponentials of the regression parameter estimates. **a**Models include only explanatory variables with *p* < 0.1. *p < 0.05, **p < 0.01, ^#^*p* < 0.001.										

## Discussion

In this study, chlorpyrifos, chlorthal-dimethyl, iprodione, phosmet, and simazine concentrations were higher in residences with nearby agricultural pesticide use than in residences without nearby agricultural use as determined by the CPUR and CROP metrics. In general, estimated agricultural pesticide use within a 1,250-m radius of residences was more strongly correlated with concentrations of pesticides in carpet dust than was estimated use within a 500-m radius for all pesticides except simazine, and estimated agricultural pesticide use during the previous 730 days was more strongly correlated with carpet dust concentrations than was use during shorter time periods for most pesticides except diazinon and phosmet (180 days) and iprodione (365 days). Previous studies found that concentrations of pesticides in carpet dust were higher in residences within 200 feet of an orchard compared with residences farther away (Fenske et al. 2002; Lu et al. 2000; Simcox et al. 1995) and in farm compared with nonfarm homes (Curwin et al. 2005; Obendorf et al. 2006). Ward et al. (2006) found that herbicide concentrations in carpet dust increased with crop acreage within 750 m of residences in Iowa and concluded that future studies should evaluate acreage beyond 750 m. A recent analysis of house dust samples from Salinas, California, that used CPUR data found that chlorpyrifos, chlorthal-dimethyl, and iprodione concentrations were 83%, 19%, and 49% higher in association with a 1-kg increase in use reported during the previous 30–180 days within a 9-square-mile area of the residence (Harnly et al. 2009).

The field dissipation half-life of these pesticides, an estimate of the overall rate of disappearance of pesticides from treated fields, ranges from 7 days for diazinon and iprodione to 89 days for simazine (U.S. Department of Agriculture 2009). For most pesticides evaluated, we found that concentrations in carpet dust were more highly correlated with reported agricultural use over the previous 2 years than over the previous year or 6 months. Further research is needed to identify important determinants of agricultural pesticide transport.

We found that concentrations of chlorpyrifos and simazine were higher in residences where a household member had potential occupational pesticide exposure. Evidence of take-home exposures from agricultural workers has been observed for pesticides measured in carpet dust, in vehicle dust, and on shoes or clothing (Bradman et al. 2007; Curl et al. 2002; Curwin et al. 2005; Harnly et al. 2009; McCauley et al. 2003). Levels of chlorpyrifos in homes treated for fleas and ticks and levels of diazinon and phosmet in homes with exterior professional treatment during the previous 12 months were higher than concentrations in homes that did not report these treatments. A multicenter study in Detroit, Michigan; Los Angeles, California; Seattle, Washington; and Iowa (Colt et al. 2004) found higher levels of chlorpyrifos and diazinon in carpet dust from homes with self-reported pesticide treatments for crawling insects, higher concentrations of carbaryl with treatment for flea and ticks, and a similar range of *R*^2^ values (0.11–0.15) as in our study. An evaluation of concentrations of polycyclic aromatic hydrocarbons in carpet dust samples from the Northern California Childhood Leukemia study also found that the best-fitting regression models explained about 15% of the variability, similar to our results for pesticides (Whitehead et al. 2009).

Our analyses are based on one carpet dust sample collected from each residence. Studies with repeated measurements of chlorpyrifos in dust taken from the same residences have found that the concentrations within a residence are correlated over time, indicating that one measurement provides a fairly reliable estimate of potential exposure (Egeghy et al. 2005; Pang et al. 2002; Wilson et al. 2009). However, there is a need to evaluate concentrations of other pesticides in repeated dust samples to determine the variability over time. Although our results are based on one sample from each residence, we found that diazinon concentrations decreased with the year of sample collection, which is likely related to the phase-out of all residential uses of diazinon in 2004 (U.S. Environmental Protection Agency 2008) and agrees with a recent study in which children’s estimated dose of diazinon decreased longitudinally over time (Wilson et al. 2009).

The main strengths of this study include the availability of reported agricultural pesticide use data and detailed crop maps near study residences, which combined with measured concentrations of pesticides in carpet dust samples from residences allowed us to evaluate the reliability of exposure classification in the context of epidemiologic studies. We were also able to include self-reported home and garden pesticide use as well as potential occupational pesticide exposure in our models to adjust for sources other than nearby use of agricultural pesticides.

Our study had several limitations, including the relatively small sample size. We used a single agricultural land use map created near the time of sample collection to calculate our CROP-based exposure metric, which may not have matched actual crop locations over the previous 2 years for more frequently rotated crops. Because we mapped crops within 1,250 m of each residence, we could not determine the impact of agricultural pesticide use on fields at greater distances. Another limitation is that home and occupational pesticide use questionnaire data assessed use only during the prior year, and participants may not have been able to accurately recall pesticide use during this time period. The residences we selected for this study were in agricultural areas and are not likely to be representative of the general population. Concentrations of pesticides in carpet dust are also not a direct measure of total pesticide exposure.

Our results suggest that some pesticides may travel more than 500 m from treated fields, and this should be considered in future exposure and epidemiologic studies. In an earlier evaluation, we found that agreement between CPUR and CROP metrics differed depending on the spatial extent evaluated (Nuckols et al. 2007). Although our results in this study were generally similar using either metric, pesticide concentrations were generally higher in homes classified as exposed by the CROP metric compared with the CPUR metric, and regression models with the CROP metric explained 2–4% more variability in concentrations of pesticides in carpet dust than did those using CPUR data alone. There is also evidence that incorporating wind direction can greatly improve the amount of variability in pesticide dust concentrations explained by regression models (Nuckols et al. 2008; Riggs 2007). To incorporate wind direction into GIS-based exposure metrics, more detailed information is needed on crop location than that provided by the CPUR data.

## Conclusions

Residences with reported use of agricultural pesticides nearby had significantly higher concentrations of pesticides in carpet dust compared with residences without nearby agricultural use for five of the seven agricultural pesticides we evaluated. We observed moderate correlations between concentrations of pesticides in carpet dust and nearby agricultural pesticide use for the same five pesticides. We observed similar correlation coefficients using either CPUR data alone or combined with crop maps (CROP metric), but *R*^2^ values were greater and modeled associations between agricultural pesticide use and pesticide concentrations in carpet dust were stronger when we estimated agricultural use based on the CROP metric.
